# Supplementary material for: Using whole blood cultures in interferon gamma release assays to detect Mycobacterium tuberculosis complex infection in Asian elephants (Elephas maximus)
Source: PLoS One. 2023 Jul 27;18(7):e0288161. doi: 10.1371/journal.pone.0288161 (PMC10374124; doi:10.1371/journal.pone.0288161)
Supplement: S1 Table — (PDF) [file pone.0288161.s001.pdf]

S1 Table. List of elephants and the amounts of eIFNγ detected from PBMC culture

PBMC Culture							
No.	sex	age	eIFNγ (pg/ml)				TB status
			Unstim	ConA	ESAT6	CFP10	
1	F	34	ND	0.577	0.469	0.758	**MP**
2	F	70	ND	0.622	0.000	0.658	**MP**
3	F	20	ND	1.140	0.000	0.902	**MP**
4	F	16	ND	1.009	0.725	0.817	**MP**
5	F	34	ND	1.328	0.782	1.305	**MP**
6	F	19	ND	0.557	0.634	0.702	**MP**
7	F	10	ND	0.733	ND	ND	**N**
8	F	31	ND	0.451	ND	ND	**N**
9	F	46	ND	1.012	ND	ND	**N**
10	F	60	ND	0.937	ND	ND	**N**
11	M	31	ND	0.862	ND	ND	**N**
12	F	80	ND	1.201	ND	ND	**N**
13	F	21	ND	0.519	ND	ND	**N**
14	F	45	ND	0.732	ND	ND	**N**
15	F	7	ND	0.597	0.680	0.689	**MP**
16	F	31	ND	0.693	0.702	0.822	**MP**
17	M	6	ND	0.469	ND	0.267	**MP**
18	M	7	ND	0.672	ND	ND	**N**
19	F	42	ND	0.496	ND	ND	**N**
20	F	47	ND	0.438	ND	ND	**N**
21	F	45	ND	0.787	ND	0.191	**MP**
22	F	45	ND	0.883	0.832	0.878	**MP**
23	F	35	ND	0.467	0.368	ND	**MP**
24	F	48	ND	0.330	0.257	ND	**MP**

*ND=not detectable
