# Supplementary material for: Using whole blood cultures in interferon gamma release assays to detect Mycobacterium tuberculosis complex infection in Asian elephants (Elephas maximus)
Source: PLoS One. 2023 Jul 27;18(7):e0288161. doi: 10.1371/journal.pone.0288161 (PMC10374124; doi:10.1371/journal.pone.0288161)
Supplement: S2 Table — (PDF) [file pone.0288161.s002.pdf]

S2 **Table .** List of elephants and the amounts of eIFNγ detected from whole blood culture

Whole Blood Culture							
No.	sex	age	eIFNγ (pg/ml)				TB status
			Unstim	ConA	ESAT6	CFP10	
1	F	34	ND	1.974	2.865	2.695	**MP**
2	F	70	ND	3.879	3.935	2.221	**MP**
3	F	20	ND	1.010	1.218	1.506	**MP**
4	F	16	ND	1.240	1.604	0.981	**MP**
5	F	34	ND	0.649	ND	0.631	**MP**
6	F	19	ND	1.630	ND	1.644	**MP**
7	F	10	ND	1.983	ND	ND	**N**
8	F	31	ND	1.687	ND	ND	**N**
9	F	46	ND	1.508	ND	ND	**N**
10	F	60	ND	2.231	ND	ND	**N**
11	M	31	ND	1.682	ND	ND	**N**
12	F	80	ND	0.624	ND	ND	**N**
13	F	21	ND	1.769	ND	ND	**N**
14	F	45	ND	1.366	ND	ND	**N**
15	F	7	ND	0.582	ND	0.743	**MP**
16	F	31	ND	3.732	4.362	4.831	**MP**
17	M	6	ND	2.027	ND	1.085	**MP**
18	M	7	ND	0.967	ND	ND	**N**
19	F	42	ND	1.385	ND	ND	**N**
20	F	47	ND	2.054	ND	ND	**N**
21	F	45	ND	1.283	0.233	ND	**MP**
22	F	45	ND	0.404	ND	0.330	**MP**
23	F	35	ND	0.574	ND	1.028	**MP**
24	F	48	ND	3.184	2.303	2.165	**MP**

*ND=not detectable
