# Supplementary material for: Using whole blood cultures in interferon gamma release assays to detect Mycobacterium tuberculosis complex infection in Asian elephants (Elephas maximus)
Source: PLoS One. 2023 Jul 27;18(7):e0288161. doi: 10.1371/journal.pone.0288161 (PMC10374124; doi:10.1371/journal.pone.0288161)
Supplement: S3 Table — (PDF) [file pone.0288161.s003.pdf]

S3 Table. List of elephants and the amounts of eIFNγ detected from mitogen stimulation

Whole Blood Culture						
No.	sex	age	eIFNγ (pg/ml)			
			Unstim	ConA	PWM	ConA+PWM
2	F	70	ND	ND	3.879	3.818
8	F	31	ND	ND	1.398	1.391
13	F	21	ND	ND	1.995	1.958
25	F	3	ND	ND	1.163	1.327
26	F	16	ND	ND	1.637	1.586
27	F	10	ND	ND	2.950	2.771
28	F	13	ND	ND	1.306	1.148
29	F	15	ND	ND	2.375	2.139
30	F	33	ND	ND	2.048	2.037
31	M	N/A	ND	ND	0.270	0.269

*ND=not detectable
