# Supplementary material for: Using whole blood cultures in interferon gamma release assays to detect Mycobacterium tuberculosis complex infection in Asian elephants (Elephas maximus)
Source: PLoS One. 2023 Jul 27;18(7):e0288161. doi: 10.1371/journal.pone.0288161 (PMC10374124; doi:10.1371/journal.pone.0288161)
Supplement: S1 File — (PDF) [file pone.0288161.s004.pdf]

S1 File

Detection of MTBC infection by Digital PCR (dPCR)

DNA preparation from elephant swab

Elephant trunk swab samples (No.1 and No.24) were collected with sterile cotton buds. The swabs samples were maintained in capped tube with normal saline solution, kept frozen until DNA preparation processed. To extract DNA from trunk swab samples, thawed the frozen samples, discarded the cotton swabs, centrifuged, and collected the pellets for DNA preparation. Total DNA from elephant trunk swab samples were prepared using Qiagen DNeasy Blood and Tissue Kit (Qiagen, Germany) according to the “Protocol: Purification of Total DNA from Animal Blood or Cells [Spin-Column Protocol]” as described by the manufacturer.

Detection of MTBC infection by Digital PCR (dPCR)

DNA preparation from elephant swab

Elephant trunk swab samples (No.1 and No.24) were collected with sterile cotton buds. The swabs samples were maintained in capped tube with normal saline solution, kept frozen until DNA preparation processed. To extract DNA from trunk swab samples, thawed the frozen samples, discarded the cotton swabs, centrifuged, and collected the pellets for DNA preparation. Total DNA from elephant trunk swab samples were prepared using Qiagen DNeasy Blood and Tissue Kit (Qiagen, Germany) according to the “Protocol: Purification of Total DNA from Animal Blood or Cells [Spin-Column Protocol]” as described by manufacturer.

Droplet Digital PCR (ddPCR)

To detect *Mycobacterium tuberculosis* complex (MTBC) specific DNA sequences in the samples, *IS6110* and *gyrB*, droplet digital PCR (ddPCR) using QX200 system (BioRad, USA) was performed as described previously (Ushio et al 2016). Primers and probe specific to *IS6110*

(IS6110 forward: 5’-GGCGTACTCGACCTGAAAGA-3’; IS6110 reverse: 5’-CTGAACCGGATCGATGTGTA-3’; IS6110 probe: 5’-[FAM]-CCACCATACGGATAGGGGAT-[BHQ-1]-3’) and *gyrB* (gyrB forward: 5’-AAGGACCGCAAGCTACTGAA-3’; gyrB reverse: 5’-GTGTTGCCCAACTTGGTCTT-3’; gyrB probe: 5’-[FAM]-ACCTCACCGGTGACGATATC-[BHQ-1]-3’), were ordered from BioRad as 20x premixed solution. The ddPCR reaction mixture of 20 µl contained 10 µl of 2x QX200 ddPCR Supermix for Probes (no dUTP), 1 µl of 20x premixed probe/primer (Final conc. 900 nM primers, 250 nM probe), and 9 µl of DNA template. Droplet were generated and transferred to for 96-well plate for PCR in a thermal cycler with the following conditions (for both *IS6110* and *gyrB*): 1cycle at 95°C for 10 min, 40 cycles at 94°C for 30 sec followed by 54°C for 90 sec, and 1 cycle at 98°C for 10 min, final hold at 4°C for infinite. After PCR amplification, the plate containing the droplets placed in the QX200 Droplet reader to measure the fluorescence signal and the results were analyzed using QuantaSoft software v.7.1.

Results

Elephant No.1 and No.24 showed positive for amplification of specific genes for MTBC by ddPCR. The copy number per 20 µl reaction mixtures of *IS6110* and *gyrB* were 186 and 15.8 copies for elephant No.1 and were 2.8 and 1.4 for elephant No.24, respectively.

(A)

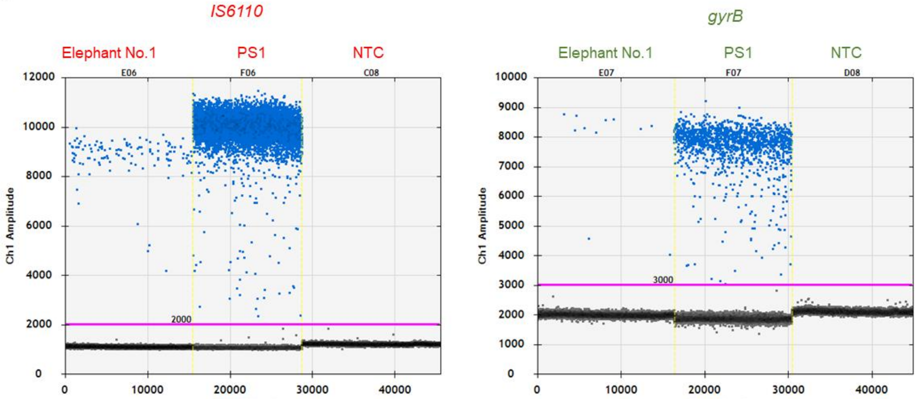

Well	Sample	Target	Concentration (copies/uL)	CopiesPer20uLWell	Copies/uLof sample (add 9uL/well)
E06	Ele No.1	*IS6110*	9.3	186	21
F06	PS1	*IS6110*	1207	24140	2682
C08	NTC	*IS6110*	0	0	0
E07	Ele No.1	*gyrB*	0.79	15.8	2
F07	PS1	*gyrB*	126	2520	280
D08	NTC	*gyrB*	0	0	0

(B)

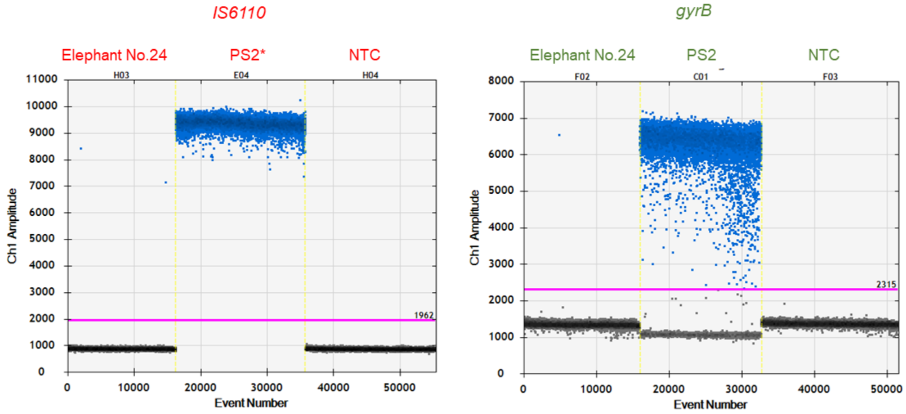

Well	Sample	Target	Concentration (copies/uL)	CopiesPer20uLWell	Copies/uL of sample (add 9uL/well)
H03	Ele No.24	*IS6110*	0.14	2.8	0.31
E04	PS2* 1:1000	*IS6110*	Saturated (overload template)		
H04	NTC	*IS6110*	0	0	0.00
F02	Ele No.24	*gyrB*	0.07	1.4	0.16
C01	PS2 1:1000	*gyrB*	2427	48540	5393.33
F03	NTC	*gyrB*	0	0	0.00

→ X dilution factor 1,000 => 5,393,330 copies/uL of sample

Plots of FAM fluorescence of insertion sequence 6110 (*IS6110*), and gyrase subunit B (*gyrB*), using DNA templates from trunk swabs of elephant No.1 and No.24 in ddPCR reactions. Each droplet from a sample was plotted on the graph represented the fluorescence intensity. Positive droplets shown in blue are above the threshold (pink solid line), all below the red line shown in black are scored negative. Positive samples (PS1 and PS2) and No Template Control (NTC) were included in each run. (A) Elephant No.1: Plots of FAM fluorescence intensity of *IS6110* (Left), *gyrB* (Right), and the table with summary results of copy number of both target genes. (B)

Elephant No.24: Plots FAM fluorescence intensity of *IS6110* (Left), *gyrB* (Right), and the table with summary results of copy number of both target genes. Copy number of PS2* (Positive sample) of *IS6110* could not be quantified due to overloading of template.

As shown in Figure above, elephant No.2 and No.24 showed positive for amplification of specific genes for MTBC by ddPCR. The copy number per 20 μl reaction mixtures of *IS6110* and *gyrB* were 18.6 and 15.8 copies for elephant No.1 and were 1.4 and 1.46 for elephant No.24, respectively.
